# Advanced Computational Methods for Radiation Dose Optimization in CT

**DOI:** 10.3390/diagnostics14090921

**Published:** 2024-04-29

**Authors:** Shreekripa Rao, Krishna Sharan, Srinidhi Gururajarao Chandraguthi, Rechal Nisha Dsouza, Leena R. David, Sneha Ravichandran, Mubarak Taiwo Mustapha, Dilip Shettigar, Berna Uzun, Rajagopal Kadavigere, Suresh Sukumar, Dilber Uzun Ozsahin

**Affiliations:** 1 Department of Radiotherapy and Oncology, Manipal College of Health Professions, Manipal 576104, Indiarechal.nisha@manipal.edu (R.N.D.); 2 Department of Radiotherapy and Oncology, Kasturba Medical College and Hospital, Manipal 576104, India; tk.sharan@manipal.edu (K.S.); shrinidhi.gc@manipal.edu (S.G.C.); 3 Department of Medical Imaging Technology, Manipal College of Health Professions, Manipal 576104, India; ldavid@sharjah.ac.ae (L.R.D.); sneha.ravichandran@learner.manipal.edu (S.R.); dilip.mchpmpl2023@learner.manipal.edu (D.S.); 4 Department of Medical Diagnostic Imaging, College of Health Sciences, University of Sharjah, Sharjah 27272, United Arab Emirates; 5 Operational Research Centre in Healthcare, Near East University, TRNC Mersin 10, Nicosia 99138, Turkey; mubarak.mustapha@neu.edu.tr (M.T.M.); berna.uzun@neu.edu.tr (B.U.); 6 Department of Biomedical Engineering, Near East University, TRNC Mersin 10, Nicosia 99138, Turkey; 7 Department of Mathematics, Near East University, TRNC Mersin 10, Nicosia 99138, Turkey; 8 Department of Radiodiagnosis and Imaging, Kasturba Medical College and Hospital, Manipal 576104, India; rajagopal.kv@manipal.edu; 9 Research Institute for Medical and Health Sciences, University of Sharjah, Sharjah 27272, United Arab Emirates

**Keywords:** computed tomography, diagnostic reference level, dose-length product, effective dose, radiotherapy

## Abstract

Background: In planning radiotherapy treatments, computed tomography (CT) has become a crucial tool. CT scans involve exposure to ionizing radiation, which can increase the risk of cancer and other adverse health effects in patients. Ionizing radiation doses for medical exposure must be kept “As Low As Reasonably Achievable”. Very few articles on guidelines for radiotherapy-computed tomography scans are available. This paper reviews the current literature on radiation dose optimization based on the effective dose and diagnostic reference level (DRL) for head, neck, and pelvic CT procedures used in radiation therapy planning. This paper explores the strategies used to optimize radiation doses, and high-quality images for diagnosis and treatment planning. Methods: A cross-sectional study was conducted on 300 patients with head, neck, and pelvic region cancer in our institution. The DRL, effective dose, volumetric CT dose index (CTDI_vol_), and dose-length product (DLP) for the present and optimized protocol were calculated. DRLs were proposed for the DLP using the 75th percentile of the distribution. The DLP is a measure of the radiation dose received by a patient during a CT scan and is calculated by multiplying the CT dose index (CTDI) by the scan length. To calculate a DRL from a DLP, a large dataset of DLP values obtained from a specific imaging procedure must be collected and can be used to determine the median or 75th-percentile DLP value for each imaging procedure. Results: Significant variations were found in the DLP, CTDI_vol,_ and effective dose when we compared both the standard protocol and the optimized protocol. Also, the optimized protocol was compared with other diagnostic and radiotherapy CT scan studies conducted by other centers. As a result, we found that our institution’s DRL was significantly low. The optimized dose protocol showed a reduction in the CTDI_vol_ (70% and 63%), DLP (60% and 61%), and effective dose (67% and 62%) for both head, neck, and pelvic scans. Conclusions: Optimized protocol DRLs were proposed for comparison purposes.

## 1. Introduction

In medicine, computed tomography (CT) scans have emerged as a crucial and beneficial tool. Day by day, the frequency of CT examinations increases [1,2,3]. Other than for diagnostic purposes, oncology patients use CT imaging to achieve personalized treatment planning. According to the WHO (World Health Organization), radiation oncology can benefit almost half of all cancer patients [4]. CT scanners dedicated to radiotherapy scans provide quality images for precise target volume and organ delineation. Ionizing radiation safety is crucial due to the cancer risk [5]. According to the International Atomic Energy Agency (IAEA), for diagnostic medical exposure, keep the exposure of patients to the minimum necessary level to achieve the required diagnostic or interventional objectives [6].

John Damilakis et al.’s study emphasizes EuroSafe Imaging’s push for establishing local DRLs via multidisciplinary teams, data collection, and adherence to national DRLs, unless clinical reasons necessitate deviation [7]. Graciano Paulo et al.’s European study on clinical diagnostic reference levels for X-ray medical imaging reviewed 23 papers from 15 countries, highlighting varied dose descriptors and advocating for protocol standardization and optimization [8]. Van der Molen et al.’s study on CT radiation exposure in the Netherlands found that effective doses varied widely across hospitals, emphasizing the need for standardization [9].

Schegerer et al. updated the DRLs for X-ray procedures in Germany, emphasizing practical applications, lowering the DRLs, and recommending optimization strategies like local reference levels and individualized protocols [10]. Dina Husseiny Salama et al. aimed to set CT diagnostic reference levels (DRLs) in Egypt, finding variations with a lower CTDI_vol_ but higher DLP values, highlighting scan range concerns [11]. Mafalanka et al. proposed DRLs for coronary CT angiography due to dose variability, noting differences between gating methods and stressing protocol optimization [12].

The computed tomography dose index volume (CTDI_vol_) and dose-length product (DLP) are crucial metrics for optimizing radiation doses in CT scans during radiotherapy. They provide reference values for exposure but do not directly relate to patient dosages. DRLs are vital in managing patient exposure and ensuring treatment precision during CT scans [13,14,15,16]. Singh et al. conducted a study that leveraged a patient-specific three-dimensional model from DICOM MRI images, employing the Pennes bioheat transfer and Arrhenius thermal damage models to simulate the impact of heat therapy on tumors and surrounding tissues. Key findings highlight the necessity of pre-clinical modeling to enhance treatment quality and decision making, notably in planning and risk assessment. It quantifies the effects of the heat deposition rate, exposure duration, and blood perfusion on tumor ablation, suggesting that treatment margins of less than 5 mm are optimal to avoid excessive damage to adjacent tissues. The research underlines the importance of accurate geometric models to evaluate treatment parameters and minimize risks related to thermal coagulation zones, thereby preserving healthy tissue and improving clinical outcomes. This study supports developing tailored, effective treatment strategies in clinical settings [17].

The present protocol provided by the manufacturer, which is used in our hospital, is a tube voltage of 120 kVp and 300 mAs; the DLP for the head and neck is 790.65 mGy.cm; and the effective dose for the head and neck is 2.45179 mSv. The DLP for the pelvis is 999.7 mGy.cm, and the effective dose for the pelvis is 15.48 mSv. These values represent the 75th percentile standards. The CTDI_vol_ value for the same scans is 17.6 mGy [18]. Patient exposure from radiotherapy CT simulations has not been a concern so far. Information on the dosage levels from radiotherapy (RT) CT simulations is limited. Despite these concerns, it is widely known that absorbed doses from CT procedures for radiation oncology patients are several orders of magnitude lower than the total absorbed dose from radiation oncology treatment. However, it is important to remember that radiation oncology CT treatments come in non-therapeutic doses and are subject to the “As Low As Reasonably Achievable” (ALARA) standard. CT scan radiation exposure varies based on protocols, patient sizes, and scanner types. The typical dose ranges from 1 to 10 mSv. Risks include cataracts, skin damage, and fetal exposure. Research on reference levels for radiation oncology CT is crucial due to potential risks and differing dose standards [19,20]. Recent prospective observational research has shown that it is possible to reduce the CT dose by more than 50% without sacrificing image quality [21].

Singh, 2024 [22] proposes a modified Pennes bioheat equation to consider tumor blood perfusion variations, improving temperature predictions for thermal therapies and treatment planning. Advanced imaging-derived perfusion coefficients aid in assessing heterogeneity, enhancing our understanding, and optimizing cancer treatments.

In radiotherapy planning, we use digitally reconstructed radiographs (DRRs) generated by CT scans to contour tumors and other relevant critical organs. The diverse purposes of diagnostic and radiotherapy imaging also require different standards for image quality [14]. CT scans provide crucial organ and tissue information. Diagnostic imaging has higher resolution needs than radiotherapy planning. Absorbed dose calculations in treatment planning systems (TPSs) use CT-derived data to optimize doses for target volumes and OARs, minimizing risks like induced carcinogenesis in non-target body areas [23,24]. CBCT and EPID imaging for patient position verification in radiotherapy can increase total radiation exposure. To minimize the impact, use appropriate protocols, limit the imaging frequency, use low-dose protocols, and optimize device positions. Doses outside the treatment area should be considered [25]. The risk associated with this imaging should be kept as low as reasonably possible because patients with primary cancer who survive can expect to live a long time.

The studies found that diagnostic reference levels can be used to optimize the radiation dose in diagnostic procedures [26,27]. However, there are limited studies on the survey of diagnostic CT radiation doses in radiotherapy treatment. 

This study aimed to develop optimized protocols for head, neck, and pelvic cancer radiotherapy CT scans and compare them qualitatively and quantitatively. It addressed the need for reduced patient doses while maintaining adequate image quality, aligning with international organizations’ calls for establishing diagnostic reference levels (DRLs). The absence of such DRLs in India underscores the importance of research to optimize radiation doses for safe and effective radiotherapy planning.

DRLs were calculated for head, neck, and pelvic cancer radiotherapy CT using present and optimized protocols compared with global studies. Before the patient scans, Catphan 503 phantom scans were used to evaluate the CT system performance across imaging parameters, ensuring optimal protocol usage for the RT CT scans [28,29].

Catphan 503 is an acrylic phantom with inserts simulating different tissues for evaluating CT system performance, including spatial resolution, low-contrast detectability, CT number accuracy, geometric distortion, and artifact reduction, making it a valuable tool in optimizing CT systems in medical imaging.

After the subjective and objective analysis of those images of the phantom, the protocol was used to perform radiotherapy CTs for head, neck, and pelvic cancer patients. The different sets of patients were used for two different phases of scanning in this study. Utilizing a phantom in a clinical scan setting, Brunner et al. measured the dose and assessed image quality to carry out an objective image analysis using metrics like noise, the contrast-to-noise ratio (CNR), and the signal-to-noise ratio (SNR) [26]. This study aimed to optimize CT scan protocols by evaluating scan parameters’ effects on image quality and dose. The SNR and FOM guide optimal protocols (kVp, mAs, and IR) based on the CNR and CTDI_vol_. The results establish radiation doses in radiotherapy CT and radiotherapy planning reference levels (RPRLs) are proposed for comparison [30].

## 2. Materials and Methods

### 2.1. CT Simulators

The Brilliance 16 Big bore CT from the Philips Medical System was used for the radiotherapy CT scans. Its features included an 85 cm bore size, a 60 cm true SFOV, 16 slices per revolution, oncology-specific tools, respiratory-correlated gating, and a patient couch assembly meeting oncology accuracy standards. Data were collected from head, neck, and pelvic scans using specific protocols.

### 2.2. Data Collection

This study included cancer patients aged 18 and above undergoing CT scans for the head, neck, and pelvic regions. The data collected encompassed various CT acquisition parameters but excluded scout images. Patients within a weight range of 40–80 kg (mean: 60 kg +/− 5 kg) were considered to avoid potential impacts on treatment planning due to varying image clarity. In the head and neck RTCT scans, the length from the vertex of the skull to the carina was considered; for the pelvis, the examination’s length was from the thoracic spine D10–D12 region to the mid-femur bone. Ethical approval was obtained from the Institutional Review Committee of Kasturba Hospital Manipal, with the project number 925/2018 approved on 10/12/2018.

### 2.3. Data Analysis

The images collected herein were analyzed both objectively and subjectively. This was a cross-sectional study conducted in the institution on head, neck, and pelvic region cancer patients. 

Optimizing the CT radiation dose involved acquiring phantom images with varied kV and mAs settings, and then reconstructing and reviewing them with oncologists and medical physicists. The optimal parameters obtained from the phantom data informed the optimized protocols for patient scans, ensuring consent and blinded analysis for spatial resolution and structure delineation quality assessment by oncologists.

Also, the obtained CT images were analyzed for subjective and objective image quality, overall image, and radiation dose evaluation. 

#### 2.3.1. Subjective Image Quality

The quality evaluation of CT images was carried out as per the current guidelines on computed tomography quality criteria [15,30]. Three experienced oncologists independently reviewed the CT images on a contouring workstation, evaluating image noise and therapeutic feasibility. Critical organs’ visibility in the head, neck, and pelvic regions was assessed using a 5-point scale: 1 = cannot identify, 2 = suboptimal, 3 = acceptable, 4 = better than acceptable, and 5 = excellently visualized.

#### 2.3.2. Objective Image Quality

Objective image quality assessment involved analyzing the signal-to-noise and contrast-to-noise ratios. The ROIs were manually placed on organs of various densities (e.g., the hippocampus, eyeballs, and CSF for the head; the bladder, muscle, fat, and femur bone for the pelvis) in CT slices, ensuring 20–30 mm^2^ ROIs for accurate measurements of tissue uniformity.

The following equation was used to assess the SNRs and CNRs [26]:

SNR = (CT ROI)/SD ROI

CNR = (CT ROI − CT Background)/SD Background

The overall evaluations of the images and radiation dose were conducted as follows: 

The figure of merit (FOM) quantifies the CT system performance by considering sensitivity, spatial resolution, and noise. It is calculated as the ratio of the CNR squared to the dose, indicating the trade-off between image quality and radiation dose. Higher FOM values signify a better imaging performance at a given dose, which is crucial for protocol optimization. The equation is [26]

FOM = CNR^2^/CTDI_vol_

The FOMs of all regions of interest were examined individually.

### 2.4. Multiple Linear Regression

Multiple linear regression is a statistical method to analyze the relationships between multiple independent variables and a dependent variable. Widely used in healthcare, it helps understand and predict health outcomes based on patient data, aiding in treatment decisions and enhancing patient outcomes through predictive modeling and data analysis.

The mathematical principle behind multiple linear regression involves constructing a model that predicts the dependent variable based on the values of the independent variables. The model is constructed using a linear equation, where the dependent variable y is expressed as a weighted sum of the independent variables $(X_{1}$ to $X_{n})$, with each variable weighted by its respective coefficient $\left(\beta_{1}\;to{\;\beta }_{n}\right)$, as shown in the equation below. The goal of the modeling process is to minimize the difference between the predicted values and the actual values of the dependent variable, known as the residual.

$$y=\beta_{0}+{\;\beta }_{1}x_{1}+{\;\beta }_{2}x_{2}+\ldots +{\;\beta }_{n}x_{n}+\varepsilon$$

where

y is the outcome variable (dependent variable);
$\beta_{0}$ is the constant (intercept);
$\beta_{1}$ to ${\;\beta }_{n}$ are the coefficients or weights of the independent variables ($x_{1}$ to $x_{n}\;$);
$x_{1}$ to $x_{n}$ are the independent variables;
ε is the error term.

In our study, a simple multiple linear regression to predict the effective radiation dose on a single parameter $X_{1}$ can be represented mathematically as follows:

$$y=\beta_{0}+{\;\beta }_{1}x_{1}+\varepsilon$$

where y is the probability of the effective dose, $\beta_{0}$ is the intercept,${\;\beta }_{1}$ is the coefficient of the single independent parameter $X_{1}$, and ε is the error term.

The statistical analyses were conducted using Python, a versatile programming language well-suited for complex data analysis and machine learning tasks. Specifically, we utilized libraries such as NumPy for numerical operations, pandas for data manipulation, and scikit-learn for implementing multiple linear regression models. The implementation tools were implemented on Jupyter Notebook and were developed on a personal computer (PC) with Windows 10 Pro, an 11th Gen Intel (R) Core (TM) i7-11700KF @ 3.60 GHz (Gigahertz) 3.60 GHz processor, 64.0 GB (Gigabyte) of installed RAM (random access memory), and a 64-bit operating system.

### 2.5. Assumptions and Limitations of Multiple Linear Regression

#### 2.5.1. Assumptions of Multiple Linear Regression

- Linearity: The relationship between the independent and dependent variables must be linear. This means that the effect of each independent variable on the dependent variable is constant across the range of values for that independent variable.
- Independence: The observations must be independent of each other. This means that one observation’s value should not affect another’s value.
- Homoscedasticity: The variance in the errors should be constant across all levels of the independent variables. In other words, the variability in the errors should be the same for all values of the independent variables.
- Normality: The errors should be normally distributed. This means that the distribution of the errors should be symmetrical and bell-shaped.
- No multicollinearity: The independent variables should not be highly correlated. High levels of correlation can lead to unstable estimates of the coefficients and make it difficult to interpret the results.

#### 2.5.2. Limitations of Multiple Linear Regression

- Causality: Multiple linear regression can establish correlation, not causation, as unaccounted factors may influence the dependent variable.
- Extrapolation: Valid within the range used for modeling; extrapolating beyond may yield inaccurate predictions.
- Outliers: Significant outliers can distort coefficients and predictions.
- Missing data: Incomplete data can lead to biased estimates and predictions.

## 3. Results

We imaged the phantom using various CT scanning parameters to assess effective dose and image quality changes. Based on qualitative and quantitative analysis, we selected the optimal kVp and mAs combinations while discarding others. Subsequent images were taken with the chosen parameters, and the effective doses were calculated, as shown in Table 1. 

The Onco protocol (120 kVp; 300 mAs) was used as the basis to estimate the changes in the effective dose. The quantitative analysis involved SNR and CNR calculations with varying densities, while the qualitative analysis was approved by an oncologist. An optimized protocol was prepared for head, neck, and pelvic cases after receiving patient consent.

Three oncologists reviewed the CT images for spatial resolution, structure boundary, and organ visibility. The analysis was blind, with CT parameters masked, ensuring unbiased assessments. Critical organs in the head, neck, and pelvis were evaluated for visibility, maintaining consistency and avoiding repeated CT scans for patients. The above parameters were rated on a 5-point scale, where 1 = cannot identify, 2 = suboptimal, 3 = acceptable, 4 = better than acceptable, and 5 = excellently visualized. A one-way ANOVA (analysis of variance) was conducted for the images’ qualitative analysis using the ratings provided by the three oncologists. The average values of the scores given by the clinicians for each patient were obtained to perform a one-way ANOVA with a 5% significance level. It was carried out to see whether any image parameters significantly differed from others. We found the results as follows: the assumptions of the one-way ANOVA were the normality of the observations, the independence of the observations, and the homogeneity of the observations, which were satisfied.

The *p*-values obtained from the analysis were 0.627 and 1 for the head, neck, and pelvis, respectively, which were greater than the significance level of 0.05. There was no significant difference between the images taken with different combinations of kVp and mAs for the head, neck, and pelvic scans. Finally, from the data collected, we prepared an optimized protocol for the head, neck, and pelvis, as presented in Table 2 and Table 3.

We conducted a study involving 120 head and neck patients and 95 pelvic cases to establish diagnostic reference levels (DRLs) and optimize our institution’s imaging protocols. Patient radiation doses were measured and analyzed to ensure compliance with acceptable limits. We used metrics such as the CNR, SNR, and FOM to evaluate image quality. By comparing these metrics before and after optimization, we assessed the success of our efforts in improving image quality while optimizing the radiation dose. Additionally, we compared the CTDI_vol_ and DLP data for each region to quantify the differences between present and optimized protocols. The results for the DLP, CTDI_vol_, and DRL for both the optimized and unoptimized protocols are presented in Table 4.

Table 5 and Table 6 present the statistically analyzed quantitative comparison between both protocols calculated for tissues of different densities.

This study aimed to use multiple linear regression to analyze the relationships between the average SNR, CNR, DLP, and dependent variables like the FOM and effective dose in various head regions. It sought a more efficient method to predict image quality and radiation dose, which is crucial for accurate disease diagnosis and treatment planning.

This study involved two phases: pre-intervention (Phase 1) and post-intervention (Phase 2) for the head regions. A multiple linear regression model was trained and tested using training and test data, with new data accurately predicting effective doses but showing minor variations in the FOM. Evaluation metrics like R^2^, RMSE, and MAE confirmed the model’s strong predictive performance, particularly in the eye region. Overall, the results highlight varying relationships between the independent variables (SNR, CNR, and DLP) and dependent variables (FOM and dose) across the head regions, with the eye region showing the strongest relationship due to its higher radiation sensitivity. Table 7 shows the evaluation metric for multiple linear regression in Phase 1

Table 8 presents the model’s performance, with all regions of the head achieving R^2^ values above 99.94%. These R^2^ values range from 99.76% to 99.94%, indicating that the independent variables (average SNR, average CNR, and DLP) account for a high percentage of the variation in the dependent variables (average FOM and effective dose) for each region. The model’s RMSE values range from 2683.21 to 78,517.36. The CSF region has the highest value, suggesting that the model has a higher degree of error when predicting the average FOM and effective dose in this region. The MAE values range from 29.96 to 163.38, with the CSF region also having the highest value, indicating a higher degree of error in the model’s predictions for this region. These results demonstrate that the model predicts the dependent variables for different head regions. The high R^2^ values and low RMSE and MAE values indicate that the model fits the data well and has a high level of predictive accuracy. As a result, this model can be used to predict the average FOM and effective dose for different head regions.

## 4. Discussion

In the Discussion Section, we interpret the results within the context of the research question. Furthermore, we analyze the significance of the findings, compare them with the existing literature, and explore potential explanations for the observed outcomes. This section also highlights any limitations of this study and suggests avenues for future research, contributing to the broader scientific understanding of the topic.

The “linear no-threshold model” (LNT) underscores the cautious approach to ionizing radiation exposure, positing that even low doses carry a small but non-negligible risk of cancer [31]. Regulatory bodies like the International Commission on Radiological Protection (ICRP) and the US Nuclear Regulatory Commission (NRC) adopt the LNT model to set radiation dose limits and guide protective practices [32]. Consequently, reducing radiation exposure during imaging, especially in radiotherapy CT simulations, is advised to mitigate patient risk [32]. Diagnostic reference levels (DRLs) offer benchmarks for radiation doses in specific procedures, aligning with the ALARA principle to minimize patient exposure [33]. Despite the recent introduction of CT simulation DRLs in radiotherapy at our institution, considerations like patient demographics, socioeconomic status, and cultural factors warrant attention for generalizability. Table 9 shows the comparison of the dose-length product (DLP) values across different CT machines from various locations, quantifying the mean DLP, its standard deviation, the range of values, and the number of scans assessed per machine. The data show significant variability in the DLP values, ranging from as low as 524 ± 63 mGy.cm in one Irish department CT machine to as high as 1444 mGy.cm in a Croatian department CT machine, highlighting discrepancies in radiation exposure levels across different settings. This variability is crucial as it underscores the need for standardized radiation doses to ensure patient safety and treatment efficacy. The relatively stable DLP measurements at this study’s own center (762 ± 46.7 mGy.cm across 120 samples) demonstrate a controlled and consistent application of radiation doses, serving as a potential benchmark for comparing and optimizing CT protocols. These findings suggest a pressing need for the establishment of diagnostic reference levels (DRLs) to harmonize radiation practices and reduce the risk of excessive radiation exposure in patients.

Table 10 presents the dose-length product (DLP) data from CT machines across three different locations, focusing on the mean DLP values, standard deviations, and the number of samples analyzed per machine. It shows a broad range of variability in DLP values, with the Slovenian department CT machine displaying a notably high standard deviation of ±2142.9 mGy.cm around a mean of 615 mGy.cm, based on 443 samples. This suggests a significant variation in dose delivery, which could indicate inconsistent imaging practices or varied patient demographics. In contrast, the Croatian department CT machine reports a higher, but consistent, mean DLP of 1133 mGy.cm from 30 samples, indicating potentially higher radiation doses, but with less variability. This study’s own center has a mean DLP of 898 ± 129 mGy.cm from 120 samples, reflecting more controlled and consistent radiation doses compared with the highly variable figures from Slovenia. These differences are critical as they highlight the importance of standardizing radiation doses across institutions to ensure patient safety and optimal diagnostic efficacy. This variability also underscores the necessity for establishing local and possibly international diagnostic reference levels (DRLs) to guide and harmonize radiation dosing practices effectively.

The bore size variation in CT scanners between diagnostic radiology and radiotherapy introduces complexity [32]. Larger bore sizes in radiotherapy CT scanners can potentially compromise image quality and may necessitate higher doses. Currently, there is a dearth of established radiotherapy CT reference levels, prompting a reliance on diagnostic CT exams as approximations [33]. While the computed tomography dose index (CTDI_vol_) and dose-length product (DLP) are pivotal in measuring radiation doses, optimization efforts are crucial [26]. Discrepancies between optimized protocol DRLs and published European data in Table 11 underscore the importance of tailored dose-reduction strategies [33].

Comparisons with existing studies reveal variations in radiation exposure levels for different anatomical regions, emphasizing the need for context-specific optimization [21,28]. Cancer patients, undergoing numerous CT imaging procedures, face heightened radiation exposure risks, underscoring the imperative for dose-reduction techniques. Strategies such as lowering radiation doses per scan or employing alternative imaging modalities hold promise for minimizing these risks while maintaining diagnostic efficacy [32]. 

The strength of our study was the large sample size, including 240 head and neck patients and 190 pelvic patients. However, a limitation was deriving the diagnostic reference levels (DRLs) from a single center, which may not fully represent the variability across institutions and patient populations. This may limit this study’s generalizability. To improve this research, future studies could include multiple centers and consider factors like body mass index for tailored protocols.

## 5. Conclusions

Single-center RT CT scan DRLs were proposed as a dose comparison and optimization platform. Because of the limited availability of RT CT DRLs in the Indian population, we compared our center’s study with international DRLs for both diagnostic and radiotherapy CT. When the results of other studies were compared with our center’s study, the optimized protocol values were lower. This study can be extended to other scanning regions in the future.

The clinical significance of this study lies in the establishment of diagnostic reference levels (DRLs), which facilitate the optimization of radiation doses while ensuring diagnostically acceptable image quality. The implementation of a low-dose protocol enables a substantial reduction in radiation exposure for patients undergoing radiology-computed tomography (RTCT).

## Figures and Tables

**Table 1:** Relation between the effective dose and CT parameters on phantom.

CT Parameters				ED	Low-Density Bone Equivalent Material		High-Density Bone Equivalent Material	
kVp	mAs	CTDI_Vol_ (mGy)	DLP	% Reduction	SNR	CNR	SNR	CNR
90	250	6.52	205.5	63.29	43.73	266.18	152.7	371.1
90	300	7.83	222.6	55.91	43.72	249.21	172.69	346.84
90	350	9.13	259.7	48.59	45.94	258.76	190.22	376.55
120	150	8.88	252.5	50.00	46.07	259.26	170.62	356.29
120	200	11.84	336.7	33.33	44.71	244.12	168.44	379.39
120	250	14.8	420.9	16.67	47.19	266.84	160.96	378.08
120	300	17.76	505.1	0.00	51.01	280.6	200.05	399.58
140	100	8.72	248	50.90	45.06	266.29	185.88	374.33
140	150	13.08	372	26.35	48.55	270.04	198.2	384.93

**Table 2:** Optimized CT protocol for head and neck.

Rotation time	0.5 s/1 s
Slice thickness	2.5 mm
Pitch	0.813
mAs	90
kVp	200

**Table 3:** Optimized CT protocol for the pelvis.

Rotation time	0.5 s/1 s
Slice thickness	5 mm
Pitch	0.813
mAs	90
kVp	250

**Table 4:** Comparison of CTDI_vol_, mean, and median values of DLP and DRLs of different protocols.

Protocol		CTDI_vol_	Scan Length (Mean)	Scan Length (Median)	DLP (Mean)	DLP (Median)	DRL
CT H&N	Present	17.76	383.8833	388.5	762.1475	760.35	791
CT PELVIS		17.76	492.49473	501	898.135789	878.7	953.2
CT H&N	Optimized	5.22	380.453	385.2	255.554	254	285
CT PELVIS		6.52	491.996	498.265	342.3968	344.0000	370

**Table 5:** Comparison of head and neck regions with tissues of different densities in quantitative analysis.

		Protocol	Mean	Median	SD	Min.	Max.
**Caudate nucleus**	**SNR**	Present	12.48	12.43	1.40	8.41	14.97
		Optimized	7.26	7.17	2.07	3.94	13.20
	**CNR**	Present	377.23	380.24	13.69	330.65	397.0
		Optimized	199.13	200.08	24.30	147.42	244.69
	**FOM**	Present	8070.57	8155.12	595.65	6325.39	10,187.20
		Optimized	7708.64	7669.64	1844.09	4163.36	11,470.0
		**Protocol**	**Mean**	**Median**	**SD**	**Min.**	**Max.**
**Hippocampus**	**SNR**	Present	12.04	12.10	1.70	7.82	14.86
		Optimized	6.77	6.45	1.70	4.00	10.97
	**CNR**	Present	457.37	460.41	21.50	399.54	507.32
		Optimized	260.43	257.23	18.43	230.97	306.73
	**FOM**	Present	11,907.98	12,042.29	1123.14	9144.62	14,499.0
		Optimized	13,230.66	12,675.91	2318.17	10,220.44	18,024.24
		**Protocol**	**Mean**	**Median**	**SD**	**Min.**	**Max.**
**Eye**	**SNR**	Present	4.19	4.19	0.64	2.3	5.77
		Optimized	4.79	4.78	1.37	2.12	7.64
	**CNR**	Present	326.36	328.30	10.96	297.57	351.92
		Optimized	272.49	275.61	21.58	228.52	336.33
	**FOM**	Present	6017.54	6075.09	396.13	5034.33	6980.46
		Optimized	14,313.29	14,552.15	2260.61	10,004.66	21,671.05
		**Protocol**	**Mean**	**Median**	**SD**	**Min.**	**Max.**
**White matter (cerebellum)**	**SNR**	Present	10.8120	11.01	0.86	8.15	11.99
		Optimized	6.91	7.14	1.54	4.03	9.93
	**CNR**	Present	426.64	431.44	18.51	358.28	461.07
		Optimized	260.82	260.30	21.82	228.48	302.92
	**FOM**	Present	10,526.14	10,743.31	900.78	7227.99	11,976.44
		Optimized	13,123.01	12,980.91	2191.25	10,000.59	17,579.22
		**Protocol**	**Mean**	**Median**	**SD**	**Min.**	**Max.**
**CSF**	**SNR**	Present	2.11	2.14	0.45	1.2	3.03
		Optimized	2.55	2.44	0.76	0.76	4.07
	**CNR**	Present	388.53	390.32	26.27	282.45	453.06
		Optimized	289.84	276.09	47.59	225.84	409.64
	**FOM**	Present	8674.07	8690.45	1097.07	6040.92	11,698.04
		Optimized	16,524.33	14,603.11	5588.13	9771.20	32,146.53

**Table 6:** Comparison of pelvic regions with tissues of different densities in quantitative analysis.

		Protocol	Mean	Median	SD	Min.	Max.
**Kidney**	**SNR**	Present	7.74	7.93	1.46	2.89	10.76
		Optimized	5.13	4.83	2.08	2.02	10.54
	**CNR**	Present	524.02	517.3	17.25	472.72	572.44
		Optimized	282.27	279.62	17.10	256.10	332.65
	**FOM**	Present	15,478.67	15,067.52	1036.74	12,582.83	18,451.16
		Optimized	12,264.82	11,992.35	1524.27	10,060.07	16,971.78
		**Protocol**	**Mean**	**Median**	**SD**	**Min.**	**Max.**
**Bladder**	**SNR**	Present	2.01	1.80	0.75	1.01	3.98
		Optimized	2.59	2.53	1.11	1.02	4.96
	**CNR**	Present	501.14	502.07	20.27	465.33	542.30
		Optimized	272.62	268.73	13.62	254.98	309.47
	**FOM**	Present	126,392.47	126,090.20	15,597.94	100,938.15	159,450.84
		Optimized	11,427.81	11,076.78	1160.89	9971.66	14,689.52
		**Protocol**	**Mean**	**Median**	**SD**	**Min.**	**Max.**
**Muscle**	**SNR**	Present	10.35	10.16	0.97	8.91	14.69
		Optimized	5.25	5.57	1.30	2.01	7.87
	**CNR**	Present	531.36	540.36	34.36	420.80	593.55
		Optimized	278.44	276.63	12.68	256.80	312.28
	**FOM**	Present	16,034.97	16,527.50	2004.18	10,034.85	19,846.27
		Optimized	11,915.66	11,737.55	1087.33	10,114.45	14,957.77
		**Protocol**	**Mean**	**Median**	**SD**	**Min.**	**Max.**
**Fat**	**SNR**	Present	−29.53	−30.09	3.98	−37.80	−20.53
		Optimized	−24.84	−24.41	2.88	−31.90	−19.76
	**CNR**	Present	452.74	454.32	18.57	421.64	513.08
		Optimized	449.18	437.80	63.05	225.43	560.86
	**FOM**	Present	11,583.05	11,622.82	968.90	10,010.32	14,836.95
		Optimized	31,548.97	29,397.06	8652.42	7794.50	48,247.15
		**Protocol**	**Mean**	**Median**	**SD**	**Min.**	**Max.**
**Femur bone**	**SNR**	Present	20.09	19.72	2.17	15.86	24.20
		Optimized	13.13	12.61	2.24	7.92	18.84
	**CNR**	Present	538.01	537.38	17.13	503.72	594.15
		Optimized	725.59	733.69	54.82	600.87	799.68
	**FOM**	Present	16,391.30	16,411.84	1048.49	14,320.00	19,889.48
		Optimized	81,204.69	82,563.20	11,989.93	55,376.64	98,080.99

**Table 7:** The evaluation metric for multiple linear regression in Phase 1.

	R^2^%	RMSE	MAE
CSF	89.35	125,008.56	124.87
Caudate nucleus	95.58	16,037.36	34.26
Eye	99.82	249.50	6.68
Hippocampus	99.00	12,795.53	50.027
White matter	97.98	16,678.85	80.20

**Table 8:** The evaluation metric for multiple linear regression in Phase 2.

	R^2^%	RMSE	MAE
CSF	99.76	78,517.36	163.38
Caudate nucleus	99.79	7428.65	52.26
Eye	99.90	3771.87	31.82
Hippocampus	99.91	3100.31	32.34
White matter	99.94	2683.21	29.96

**Table 9:** Comparison of DRLs (DLPs) for pelvis.

Different CT Machines	Mean DLP (Standard Deviation) (mGy.cm)	Range (mGy.cm)	Number of Samples
Irish department CT 1	1146 ± 364	857–1912	10
Irish department CT 2	818 ± 54	713–878	10
Irish department CT 3	524 ± 63	429–618	10
Irish department CT 4	877 ± 259	509–1441	10
Irish department CT 5 [34]	578 ± 158	379–771	10
European department CT [35]	756 ± 107	522–913	10
Our present study center CT	762 ± 46.7	497–518	120
Slovenian department CT [25]	741.3 ± 362.5	Not published	278
Croatian department CT [24]	1444	Not published	30

**Table 10:** Comparison of DRLs (CTDI_vol_ and DLP) for H&N.

Different CT Machines	Mean DLP (Standard Deviation) (mGy.cm)	Number of Samples
Slovenian department CT [25]	615 ± 2142.9	443
Croatian department CT [24]	1133	30
Our present study center CT	898 ± 129	120

**Table 11:** Comparison of DRLs (CTDI_vol_ and DLP).

	DRL			
Reference	CTDI_vol_ in mGy		DLP in mGy.cm	
Radiotherapy planning CT	H&N	Pelvis	H&N	Pelvis
Celine Clerkin et al. [26]	21	NA	882	NA
Nika Zalokar et al. [25]	22.6	17.9	969.2	667.1
Ana Bozanil et al. [30]	35	20	1444	1133
European diagnostic CT [35]	NA	Not available	NA	500
This study	17.76	17.76	790.7	953.2

## Data Availability

Data is available if requested from the authors for a specific reason.
